# Prevalence of Domestic Violence among Infertile Women attending Subfertility Clinic of a Tertiary Hospital

**DOI:** 10.31729/jnma.4886

**Published:** 2020-06

**Authors:** Ajita Silwal, Bandana Thapa

**Affiliations:** 1 Bir Hospital Nursing Campus, National Academy of Medical Sciences, Kathmandu, Nepal.

**Keywords:** *domestic violence*, *infertility*, *violence*

## Abstract

**Introduction:**

Millions of couples suffered from Infertility worldwide. Infertility can cause intense emotional pain in women resulting in stress, anxiety and depression. Domestic violence in infertile women can further results in poor health status and lowers the quality of life. The objective of this study is to find out the prevalence of domestic violence among infertile women attending subfertility clinic of tertiary hospital.

**Methods:**

This descriptive cross-sectional study was conducted among infertile women in a tertiary hospital from July to August 2018 after taking ethical approval. Convenient sampling was used. Face to face interview was conducted using a structured interview schedule. Data analysis was done in the Statistical Package for Social Sciences. Descriptive statistics (frequency, percentage) were used to analyze the data. Point estimate at 95% CI was calculated along with frequency and proportion for binary data.

**Results:**

Domestic violence was found among 62 (55.35%) women at 95% Confidence Interval (46.15-64.55). The emotional violence accounted for 57 (50.89%), physical violence for 19 (16.96%) and sexual violence for 18 (16.07%). The prevalence of domestic violence was more 22 (61.11%) in women with secondary infertility than in women with primary infertility 40 (52.63%). The main perpetrators of domestic violence were family members 28 (45.16%).

**Conclusions:**

The study concluded that women experiencing infertility are exposed to various forms of domestic violence, emotional one being most common. Routine screening for domestic violence in infertility clinics is necessary to give affected women an opportunity to access appropriate health care and support services.

## INTRODUCTION

Childbearing is an immense pleasure for every woman. Globally there are about 48.5 million infertile couples and 14.4 million of them live in South Asia.^1^ In Nepal, although a child holds the greatest importance to the couples and families, most of the women are facing the silent tragedy of infertility.^2^ The consequences of infertility in developing countries like Nepal range from poor marital adjustment, social isolation, psychological effects, and violence.^3^

Violence, stress, anxiety, and depression occurs in a vicious cycle in infertile women that lowers the quality of life and success of treatment.^4^^–^^6^ Many of them end up committing suicide and self-injurious behavior.^7^ As violence is a major

health and human rights concern and scarcity of studies in related areas has motivated for the conduction of the study.

The present study aims to find out the prevalence of domestic violence among infertile women in a tertiary hospital.

## METHODS

A descriptive cross-sectional study was conducted in a tertiary hospital over a period of one month from July to August 2018. Ethical clearance was taken from the Institutional Review Board of National Academy of Medical Sciences and Institutional Review committee of PMWH. Data were collected from 112 women attending the subfertility clinic of PMWH after obtaining written consent.

The sample size of the study was estimated for a finite population using a confidence interval of 95%. The sample size was calculated using a standard formula.

Sample size (n) = Z^2^ × (p × q)/e^2^

                = 1.96 × 0.45 × (1-0.45)/0.05^2^

                = 381

Where,

n = required sample size
p = prevalence of domestic violence among
  Infertility women (45% from the previous study)^8^
q = 1-p
e = margin of error, 5%
Z = 1.96 at 95 % CI

In Paropakar maternity and Women’s Hospital, Population (N): 145

Adjuted Sample size = (N × n)/(N+1)(n+N-1)

                = 106

Non-response rate= 5%

Therefore the final sample size for the study was 112.

Convenient sampling technique was used in this study. Every day all the women visiting the subfertility clinic for consultation regarding infertility management were identified from the distinct subfertility OPD card as the women with other gynecological problems also visit the subfertility clinic of PMWH. Those who were willing to participate were included in the study and were invited to a private room after their consultation with the doctor. Written informed consent was obtained. Women attending Extended Hospital Service (EHS) were not included in the study.

Face to face interview was conducted with women in the absence of their spouse using a standard tool used in Nepal demographic and health survey (NDHS),2016 to assess the prevalence of domestic violence.^9^ Threaten of divorce is added to the emotional violence as literature has shown a high prevalence of it.^10,11^ Data were coded for entry and analysis into Statistical Package for Social Sciences (SPSS) version 16. Descriptive statistics (frequency and percentage) were used to describe the quantitative study variables.

## RESULTS

The results showed that among 112 women, 62 (55.35%) experienced some types of domestic violence in the last 12 months. The prevalence of domestic violence in women with primary infertility was 40 (52.63%) whereas those in secondary infertility were 22 (61.11%) (Table 1).

**Table 1:** Prevalence of domestic violence.

	Domestic violence	
Type of Infertility	Yes n (%)	No n (%)
Primary (n=76)	40 (52.63)	36 (47.36)
Secondary (n=36)	22 (61.11)	14 (38.88)
Total	62 (55.35)	50 (44.64)

Emotional violence was the most common type of violence accounting for 57 (50.89%) of the women. Likewise, 19 (16.96%) women were victims of physical violence and 18 (16.07%) of them were exposed to sexual violence. Emotional violence was found more 21 (58.33%) in women with secondary infertility than in women with primary infertility 36 (47.36). Similarly, physical 7 (19.44%) and sexual violence 6 (16.67%) were also more prevalent in women with secondary infertility (Table 2).

**Table 2:** Distribution of types of domestic violence.

	Primary	Secondary	Total
Type of domestic Violence	n (%)	n (%)	n (%)
Emotional	36 (47.36)	21 (58.33)	57 (50.89)
Physical	12 (15.79)	7 (19.44)	19 (16.96)
Sexual	12 (15.79)	6 (16.67)	18 (16.07)

The main perpetrators of domestic violence were family member only 28 (45.16%) whereas least 13 (20.97%) perpetrators were spouse only (Table 3).

**Table 3:** Perpetrators of domestic violence.

Perpetrators	n (%)
Spouse only	13 (20.97)
Family member only	28 (45.16)
Both	21 (33.87)

The Socio-demographic characteristic of women is shown. The mean age of the women was 27.44 years with SD of 5.214 years. Majority 64 (88.39%) of the women had attended SEE and higher education levels. Similarly, most 49 (43.75%) of the women were Janjati group and were housewives 71 (63.39%).

The majority of the women 67 (59.82%) belonged to a single family (Table 4).

**Table 4:** Socio-demographic characteristics of women.

Characteristics	
Age (in years)	n (%)
15-19	
20-24	1 (0.89)
25-29	35 (31.25)
30-34	39 (34.82)
35-39	23 (20.54)
40-44	11 (9.82)
Educational status	13 (11.61)
No Education	20 (17.86)
Primary Education	15 (13.39)
Secondary Education	64 (57.14)
SEE and above	
Ethnicity	15 (13.39)
Dalit	49 (43.75)
Janjati	8 (7.14)
Madhesi	1 (0.89)
Muslim	36 (32.14)
Brahmin/Chhetri	3 (2.68)
Others	
Employment Status	71 (63.39)
Housewife	41 (36.61)
Employed	
Type of Marriage	56(50.00)
Love	56 (50.00)
Arrange	
Type of family	67 (59.82)
Single	45 (40.18)
Joint	1 (0.89)

The fertility specific characteristics of women are shown. The mean age at marriage was 22±4 years. The duration of marriage was five years and less in most 73 (65.18%) of the women. In regards to the duration of infertility, the majority 57 (50.89%) of the woman were of categories more than two years (Table 5).

**Table 5:** Fertility specific characteristics.

Characteristics (in years)	n (%)
Age at marriage	
≤20	49 (43.75)
>20	63 (56.25)
Duration of marriage	
≤ 5	73 (65.18)
>5	39 (34.82)
Duration of Infertility	
≤ 2	55 (49.11)
>2	57 (50.89)

## DISCUSSION

In our study, 62 (55.35%) of women experienced some types of domestic violence in the last 12 months. The findings are also supported by another study conducted in India.^12^ However, the prevalence was found much higher in the Iranian setting.^13,14^ These differences may be due to cultural diversities in the study population as well as a different data collection tool.

In our study, violence was found higher 22 (61.11%) in women with secondary infertility in comparison to women with primary infertility 40 (52.63%). These figures are comparable to the figures obtained in Pakistan where violence on the base of gender has a similar prevalence revealing that women with no live children were more likely to be the victims of violence compared to those who had a live child.^10^ In contrary to this study, a study done in Nigeria revealed that the women who have two or more children were more likely to experience spousal violence compared with childless women.^15^

In the present study, emotional violence was most 57 (50.89%) common form of violence. In a study verbal abuse was the most common type of violence followed by intimidation for divorce. The percentage of women experiencing physical violence was 19 (16.96%) in our study. This is similar to figures recorded in studies performed in India.^12^ Unlike our findings, the study conducted in another part of India showed a higher prevalence of physical violence.^8^

The current study further illustrated the prevalence of sexual violence as 18 (16.07%) in the form of forceful sexual intercourse or sexual positions, the highest proportion being the use of physical force to have sexual intercourse. Sexual violence was much higher in a study in Iranian and Nigerian setting.^16,17^ Women hesitation to open up about sexual activities may be the reason for this contradiction.

In regards to perpetrators of domestic violence, the majority of them were family members 28 (45.16%). In a qualitative study, most of the women expressed that they are tortured by their in-laws verbally.^11^ In contrast to the study, a study done in Iran showed husband as perpetrators in all cases.^18^

This study findings are not representative of the general population as the study was conducted in a selective tertiary hospital setting. The study does not address the situation among women with male factor infertility. Domestic violence especially the physical and sexual ones may not have been expressed due to cultural or social issues like shame and embarrassment.

## CONCLUSIONS

The present study concluded that women experiencing infertility are subjected to various forms of domestic violence. Emotional violence was the most common type of violence whereas Physical and Sexual violence was nearly equally prevalent among infertile women.

Violence as being an issue of human right concern and overall quality of life of women, it is necessary to screen for violence in infertile women to identify those affected and to give these women an opportunity to access appropriate health care and support services.

## References

[1] Mascarenhas MN, Flaxman SR, Boerma T, Vanderpoel S (2012). National, Regional, and Global Trends in Infertility Prevalence Since 1990: A Systematic Analysis of 277 Health Surveys.. PLoS Med.

[2] Dangal G (2008). A study of reproductive morbidity of women in the Eastern Terai Region of Nepal.. Nepal J Obstet Gynaecol..

[3] Khanal B Exercise in fertility. The Kathmandu Post [Internet].. http://kathmandupost.ekantipur.com.np/printedition/news/2014-06-14/exercise-in-fertility.html.

[4] Awtani M, Mathur K, Shah S, Banker M (2017). Infertility stress in couples undergoing intrauterine insemination and in vitro fertilization treatments.. J Hum Reprod Sci..

[5] Rooney KL, Domar AD (2018). The relationship between stress and infertility.. Dialogues Clin Neurosci..

[6] Yusuf L (2016). Depression, anxiety and stress among female patients of infertility; A case control study.. Pakistan J Med Sci..

[7] Shani C, Yelena S, Reut BK, Adrian S, sami H (2016). Suicidal risk among infertile women undergoing in-vitro fertilization: Incidence and risk factors.. Psychiatry Research..

[8] Niranjani Jayanthi, Nishanthini (2017). Study on Domestic Violence in Infertile Women.. Int J Sci Res Publ..

[9] Ministry o Health, New ERA, Inner city Fund. 2017. Nepal Demographic and Health Survey 2016..

[10] Sami N, Ali TS (2012). Domestic violence against infertile women in Karachi, Pakistan.. Asian Rev Soc Sci..

[11] Thapa BB (2015). Lived Experience of Infertility Among.. J Nobel Med Coll..

[12] Satheesan SC, Satyanarayana VA (2018). Quality of marital relationship, partner violence, psychological distress, and resilience in women with primary infertility.. Int J community Med Public Health..

[13] Ozgoli G, Sheikhan Z, Zahiroddin A, Nasiri M, Amiri S, Badr FK (2016). Evaluation of the Prevalence and Contributing Factors of Psychological Intimate Partner Violence in Infertile Women.. J Midwifery Reprod Heal..

[14] Alijani F, Keramat A, Gardeshi ZH, Khosravi A, Afzali M, Habibi F (2018). Domestic violence among infertile women: a study in north of Iran.. Am J Exp Clin Res..

[15] Solanke BL, Bisiriyu AL, Oyedokun A (2018). Is the likelihood of spousal violence lower or higher among childless women? Evidence from Nigeria demographic and health surveys.. BMC Womens Health..

[16] Sheikhan Z, Pzgoli G, Azar M, Alavimajd H (2014). Domestic violence in Iranian infertile women.. Med J Islam Repub Iran..

[17] Iliyasu Z, Galadanci HS, Abubakar S, Auwal MS, Odoh C, Salihu HM (2016). et al. Phenotypes of intímate partner violence among women experiencing infertility in Kano, Northwest Nigeria.. Int J Gynecol Obstet..

[18] Ardabily HE, Moghadam ZB, Salsali M, Ramezanzadeh F, Nedjat S (2011). Prevalence and risk factors for domestic violence against infertile women in an Iranian setting.. International Journal of Gynecology and Obstetrics..

